# Extracellular amastigotes of *Trypanosoma cruzi* are potent inducers of phagocytosis in mammalian cells

**DOI:** 10.1111/cmi.12090

**Published:** 2013-01-09

**Authors:** Maria Cecilia Fernandes, Andrew R Flannery, Norma Andrews, Renato A Mortara

**Affiliations:** 1 Department of Cell Biology and Molecular Genetics, University of MarylandCollege Park, MD, USA; 2 Departamento de Microbiologia, Imunologia e Parasitologia, Escola Paulista de Medicina, Universidade Federal de São PauloSão Paulo, Brazil

## Abstract

The protozoan parasite *Trypanosoma cruzi*, the aetiological agent of Chagas' disease, has two infective life cycle stages, trypomastigotes and amastigotes. While trypomastigotes actively enter mammalian cells, highly infective extracellular amastigotes (type I *T. cruzi*) rely on actin-mediated uptake, which is generally inefficient in non-professional phagocytes. We found that extracellular amastigotes (EAs) of *T. cruzi* G strain (type I), but not Y strain (type II), were taken up 100-fold more efficiently than inert particles. Mammalian cell lines showed levels of parasite uptake comparable to macrophages, and extensive actin recruitment and polymerization was observed at the site of entry. EA uptake was not dependent on parasite-secreted molecules and required the same molecular machinery utilized by professional phagocytes during large particle phagocytosis. Transcriptional silencing of synaptotagmin VII and CD63 significantly inhibited EA internalization, demonstrating that delivery of supplemental lysosomal membrane to form the phagosome is involved in parasite uptake. Importantly, time-lapse live imaging using fluorescent reporters revealed phagosome-associated modulation of phosphoinositide metabolism during EA uptake that closely resembles what occurs during phagocytosis by macrophages. Collectively, our results demonstrate that *T. cruzi* EAs are potent inducers of phagocytosis in non-professional phagocytes, a process that may facilitate parasite persistence in infected hosts.

## Introduction

Foreign particles, apoptotic bodies and most invading microorganisms are eliminated from the body by phagocytic leucocytes, which play a crucial role in the innate immune response (Geissmann *et al*., 2010). The ingestion of extracellular objects, also known as phagocytosis, is receptor-mediated and actin-dependent (Aderem and Underhill, 1999). Although it is known that several other mammalian cells are capable of phagocytosis, the efficiency of the process varies greatly. To emphasize this variation in phagocytic capacity between different cell types, Rabinovitch coined the terms ‘professional’ and ‘non-professional’ phagocytes (Rabinovitch, 1995).

The unique ability of professional phagocytes to efficiently internalize a variety of targets is linked to the expression of an array of specialized phagocytic receptors such as those for immunoglobulins (Fc receptors), complement and mannose (Aderem and Underhill, 1999). The intracellular vacuoles generated in this receptor-mediated uptake process, designated as phagosomes, undergo a series of fusion and fission events that modify the composition of their limiting membrane and intravacuolar contents. This process is referred to as phagosome maturation, and grants the vacuole with degradative properties, which are central to its microbicidal function (Berón *et al*., 1995; Tjelle *et al*., 2000). Lipid-mediated signalling and phosphoinositides play an important role in this process, from the initial formation of the phagocytic cup until the final steps of phagosome maturation (Botelho *et al*., 2000; Vieira *et al*., 2001; Yeung *et al*., 2006; Botelho and Grinstein, 2011). Chimeric constructs consisting of fluorescent proteins fused to pleckstrin homology (PH) domains that recognize the headgroups of phosphatidyilinositol-4,5-bisphosphate (PI(4,5)P_2_) or phosphatidylinositol-3,4,5-trisphosphate (PI(3,4,5)P_3_) have been exploited as very useful tools to characterize the dynamics of these molecules in cells during particle uptake in macrophages (Botelho *et al*., 2000; Vieira *et al*., 2001).

The vast majority of studies on phagocytosis have been performed in primary macrophages or macrophage-derived cell lines. However, working with these cell lines imposes numerous challenges and technical barriers, which are easily overcome when working with epithelial or fibroblast cell lines. Unfortunately, the limited repertoire of particles (mainly pathogens) that can be internalized by non-professional phagocytes restricts the type of studies that can be done in these cells. While intracellular bacterial pathogens have evolved mechanisms to perturb cytoskeleton dynamics and stimulate their uptake by non-professional phagocytes (Galan and Bliska, 1996; Finlay and Cossart, 1997; Alonso and Garcia-del Portillo, 2004), their small size also renders them a poor tool for the study of phagocytosis. Larger protozoan parasites, which can be much more readily visualized by light microscopy, usually infect non-phagocytic cells by active invasion, as is the case for *Toxoplasma gondii* (Morisaki *et al*., 1995; Dobrowolski and Sibley, 1996), or the tissue culture trypomastigote stage of *Trypanosoma cruzi* (Tardieux *et al*., 1992; Fernandes *et al*., 2011b). In both instances, the host cell actin cytoskeleton does not play a major role in the host cell invasion process. Curiously, however, metacyclic trypomastigote forms of *T. cruzi* appear to have evolved a strain-dependent requirement on actin polymerization to invade mammalian cells (Ferreira *et al*., 2006).

*Trypanosoma cruzi* amastigote forms are usually found in the cytoplasm of infected cells, but can also be generated by extracellular differentiation of trypomastigotes. These extracellular amastigotes share morphological and immunochemical markers with their intracellular counterparts, and are also capable of invading and sustaining infections in mammalian cells (Nogueira and Cohn, 1976; Ley *et al*., 1988). However, differently from the infective trypomastigote stages, the extracellularly generated amastigotes (henceforth referred to as EAs) were shown to promptly aggregate actin filaments after attaching to HeLa cells (Mortara, 1991; Procópio *et al*., 1999).

EAs from group I *T. cruzi* strains (such as the G strain) were found to enter mammalian cells much more efficiently than group II (Y strain) or VI (CL strain) parasites (Fernandes and Mortara, 2004; Mortara *et al*., 2005; da Silva *et al*., 2006; Fernandes *et al*., 2006; Bambino-Medeiros *et al*., 2011; Rodrigues *et al*., 2012). Further studies revealed that EAs of either G or Y strains are capable of initiating infections in a wide range of mammalian cells and, as the flagellated trypomastigotes, elicit responses from the host cell (Fernandes and Mortara, 2004; Fernandes *et al*., 2006; Fernandes *et al*., 2007a,b; Cruz *et al*.*,* 2012). Therefore, for this study we have chosen the G strain as a model for type I *T. cruzi.*

The precise mechanism of EA internalization is not yet fully characterized. Here we describe that EAs are effective inducers of classical phagocytosis in non-professional phagocytes. We demonstrate that EA forms induce vigorous localized host cell actin polymerization during the early steps of invasion, and are capable of being engulfed by various mammalian cells at levels comparable to macrophages. Furthermore, we show that these parasites recruit to their phagocytic cup and parasitophorous vacuole the same host cell markers that were shown to be recruited during classical particle phagocytosis in professional phagocytes. This remarkable ability to induce efficient phagocytosis is likely to be a specific adaptation of these parasites to infect various cell types and propagate the infection in the mammalian host.

## Results and discussion

### *T. cruzi* EAs trigger formation of structures rich in polymerized actin which mediate their internalization in non-professional phagocytes

**Fig. 1:**
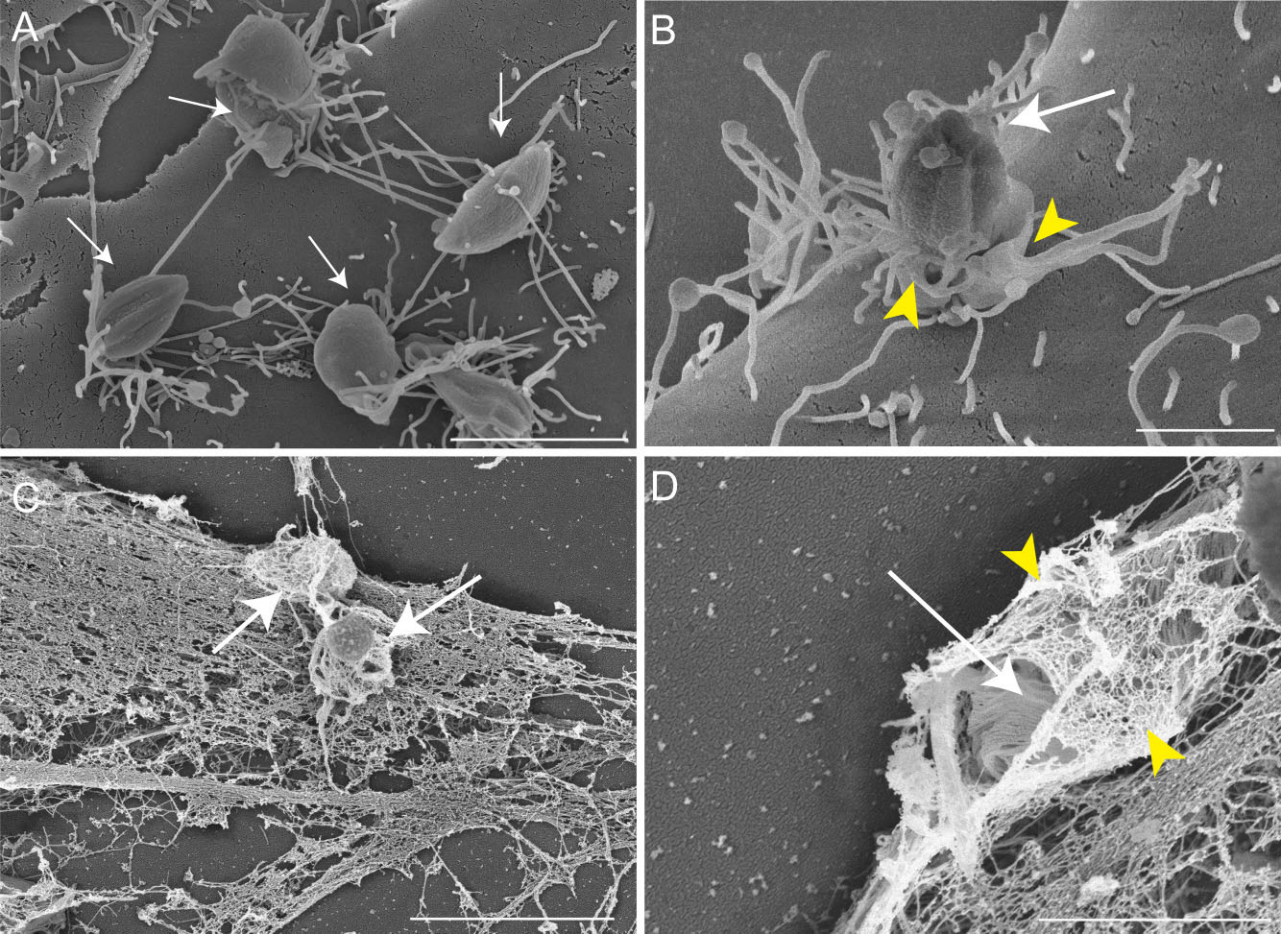
*T. cruzi* G strain EAs interact with microvilli on the surface of HeLa cells. A and B. HeLa cells and parasites imaged by Field Emission-SEM; (A) parasites (arrows) attached to surface microvilli; (B) a cup-like plasma membrane extension (yellow arrowheads) is clearly visible surrounding the parasite (arrow). C and D. Detergent-extracted cells reveal cytoskeletal elements (black arrowheads) surrounding internalized parasites (white arrows); in (D) a basket-like array of thin (5–7 nm) filaments (yellow arrowheads) is clearly visible around the parasite (white arrow). Bars: (A) 5 μm, (B) 2 μm, (C) 5 μm, (D) 2 μm.

**Fig. 2:**
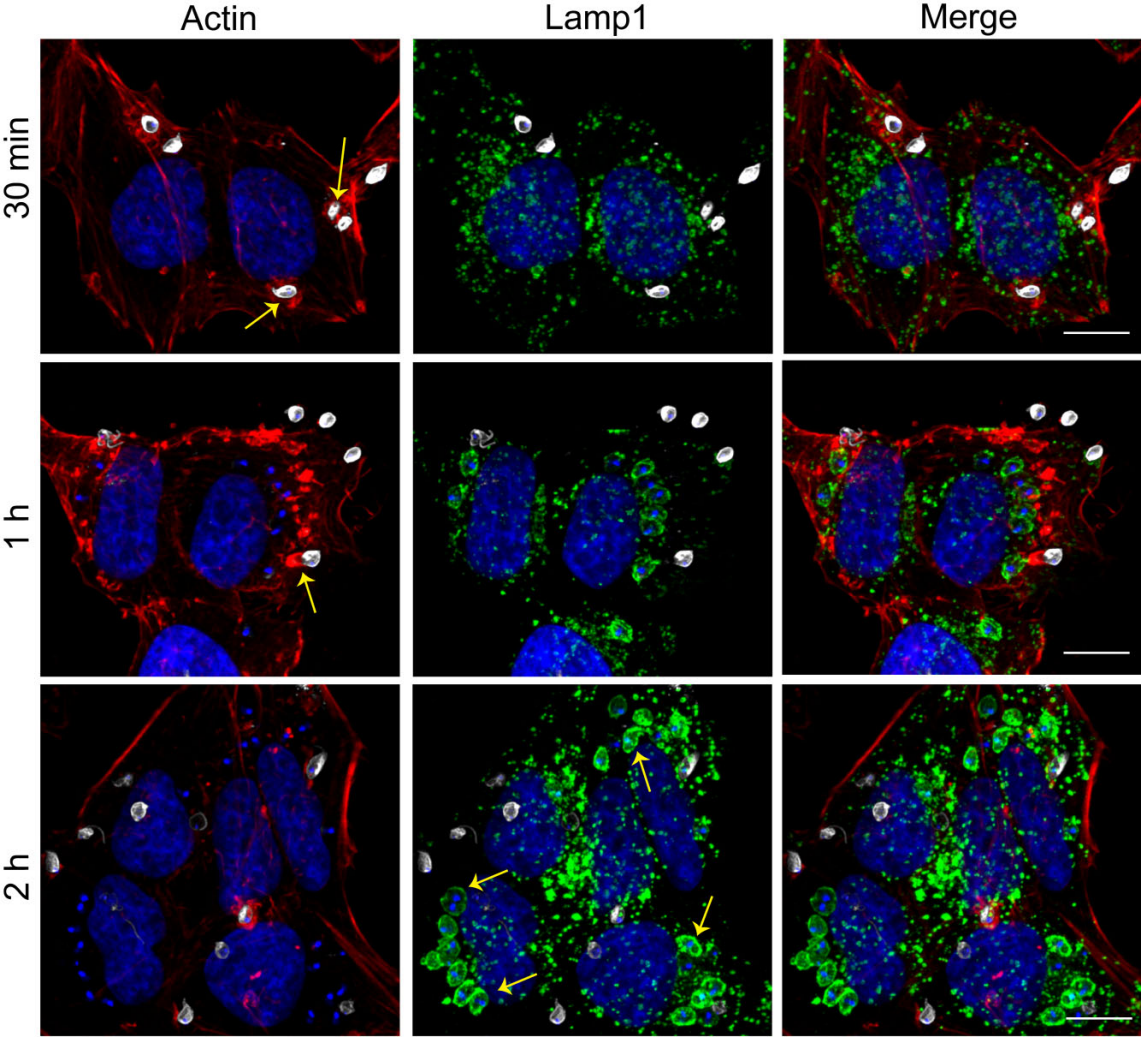
*T. cruzi* G strain EAs are internalized within actin-rich plasma membrane extensions. After 30 min (upper panels), 1 h (middle panels) and 2 h (lower panels) of infection, coverslips were fixed and processed for fluorescence microscopy. Samples were stained with phalloidin (red), anti-Lamp1 (green) and recently attached parasites (not fully internalized) were stained with anti-*T. cruzi* polyclonal (grey). The yellow arrows (30 min and 1 h) indicate actin rich structures that resemble phagocytic cups, which are more abundant at earlier time points of infection. Over time, more parasites were observed inside Lamp1 positive vacuoles (yellow arrows, 2 h).

It was previously demonstrated that *T. cruzi* EAs of the G strain (Yoshida, 1983) promptly induce actin polymerization after contact with HeLa or Vero cells (Mortara, 1991; Procópio *et al*., 1999). However, at the time of these original findings it was not clear if the process resembled what occurs during infections with enteropathogenic *Escherichia coli* (EPEC), where actin-rich pedestals are formed at the interface between the bacteria and host cells (Frankel and Phillips, 2008), or if it was more akin to phagocytosis. To examine in more detail the cytoskeletal and morphological rearrangements specifically induced by *T. cruzi* EAs, we performed field scanning electron microscopy and confocal microscopy in HeLa cells incubated with EAs without a prior centrifugation step. After 30 min of incubation, parasites were observed strongly attached to HeLa cell microvilli (Fig. 1A), and a plasma membrane extension resembling a cup-like structure was clearly visible extending upwards around the parasite (Fig. 1B). Exposure of cytoskeletal elements by detergent extraction revealed that EAs were surrounded by a dense ‘cage’ of 5–7 nm filaments (Fig. 1C and D). Confocal fluorescence images (Fig. 2) of phalloidin-stained samples demonstrated that attached parasites (white, stained with anti-*T. cruzi* antibodies prior to permeabilization) recruit polymerized actin at very early stages of the invasion process. At later points the internalized parasites were observed inside vacuoles positive for the lysosomal glycoprotein Lamp1 (green), indicating delivery of lysosomal markers as maturation progresses.

**Fig. 3:**
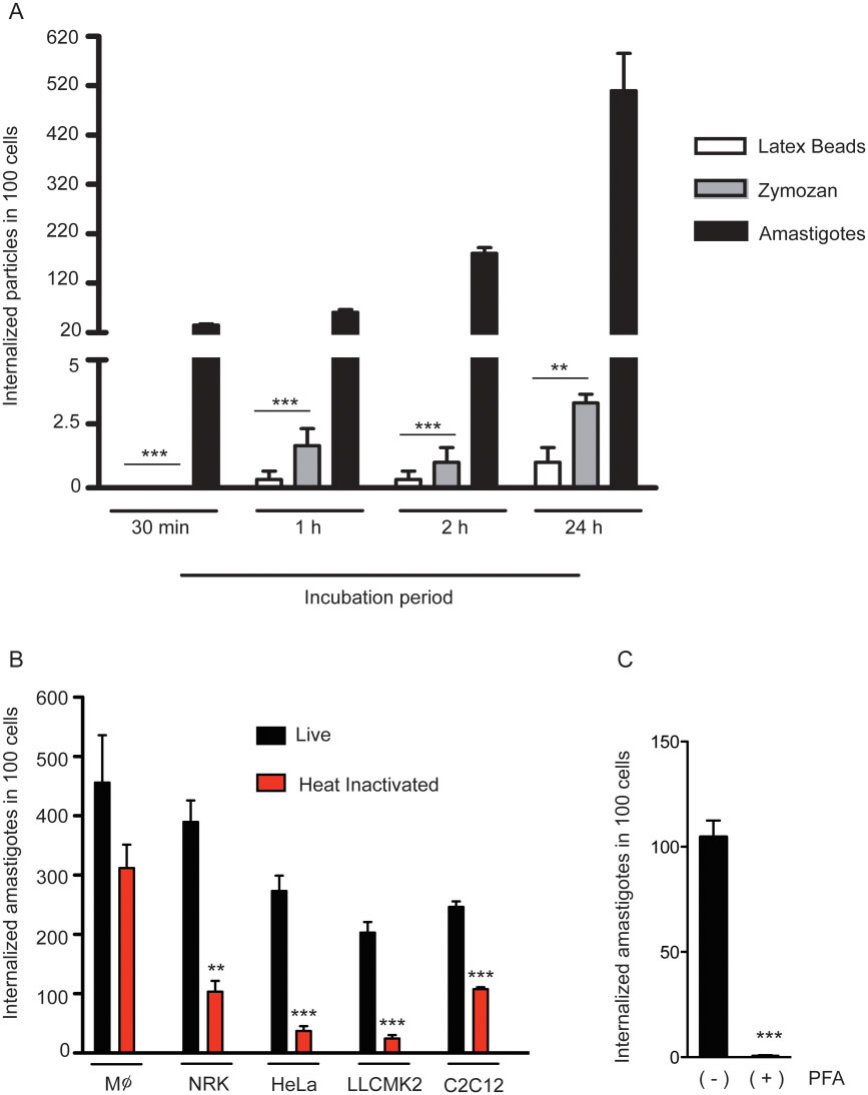
*T. cruzi* G strain EAs induce their own phagocytosis in non-phagocytic mammalian cells. A. Latex Beads (white bars), Zymosan (grey bars) and EAs (black bars) were incubated with HeLa cells for the indicated time periods. After 2 h cells were washed and new medium was added for an additional 24 h. The number of internalized particles was determined microscopically. The data correspond to mean of triplicates ± SD. ***P < 0.0002, Student's *t* test; **P = 0.0013, Student's *t* test comparing zymosan and latex bead internalization to EA internalization. B. Live (black bars) or heat-inactivated (30 min at 56°C) (red bars) EAs were incubated with the indicated mammalian cell line for 2 h, and the number of internalized parasites was determined microscopically. The data correspond to mean of triplicates ± SD. ***P < 0.0005, Student's *t* test; **P = 0.0011, Student's *t* test comparing live and heat-inactivated parasite internalization. C. EAs were treated or not with 2% PFA for 4 min, washed four times with PBS and exposed to cells for 2 h. The data correspond to mean of triplicates ± SD. ***P < 0.0005, Student's *t* test.

To directly demonstrate that the parasite was inducing host cell membrane protrusion, actin rearrangement and phagocytosis, we quantified uptake of EAs by HeLa cells in parallel with inert particles (Fig. 3A). The results clearly demonstrated that neither zymosan nor latex beads were phagocytosed by HeLa cells at levels comparable to *T. cruzi* EAs. While after 30 min there was no uptake of inert particles, we could already observe an average of about 30 internalized EAs per 100 HeLa cells. In 2 h there was a 100-fold increase in the efficiency of EA uptake, when compared with either zymosan or latex beads (Fig. 3A).

To further demonstrate the ability of EAs to induce phagocytosis in other non-professional phagocyte cell lines, we compared the number of internalized parasites, live and heat-inactivated, in bone marrow derived mouse macrophages and NRK, HeLa, LLCMK2 and C2C12 cells (Fig. 3B). The results demonstrated that live EAs were efficiently internalized by non- professional phagocytes at levels that were similar or only slightly reduced when compared with macrophages. Interestingly, heat-inactivated (Fig. 3B) or lightly fixed (Fig. 3C) parasites were significantly less internalized by the non-professional phagocyte cell lines, indicating that the efficient uptake process characteristic of *T. cruzi* EAs was induced by the live parasites. In contrast, heat-killed and live EAs were equally taken up by macrophages (Fig. 3B).

It was previously demonstrated that wortmannin, a classical inhibitor of PI3 kinase and a potent inhibitor of phagocytosis, decreases EA invasion (Fernandes *et al*., 2006). Earlier studies also showed that pre-treatment of host cells with the actin filament disrupting agent cytochalasin D also significantly impairs EA invasion (Procópio *et al*., 1998). Our results corroborate these previous findings and further demonstrate that phagocytosis can be induced by *T. cruzi* EA in several different types of non-professional phagocytic cells. The fact that only professional phagocytes were able to internalize heat-inactivated EAs at levels comparable to control parasites suggests that intact surface molecules are necessary to promote efficient uptake and to trigger efficiently the signalling cascade that leads to internalization.

These results showed that *T. cruzi* EAs trigger a markedly different invasion pathway than the flagellated tissue culture trypomastigotes (Procópio *et al*., 1998), which do not rely on host cell actin polymerization to enter host cells (Schenkman *et al*., 1991; Tardieux *et al*., 1992; Fernandes *et al*., 2011a). Trypomastigotes were shown to wound the host cell and to take advantage of the plasma membrane repair mechanism which involves lysosomal exocytosis and subsequent endocytosis (Fernandes *et al*., 2011b). Although EAs ultimately also reside in Lamp1 enriched compartments intracellularly (Procópio *et al*., 1998), the results presented in this work clearly emphasize the different invasion strategies used by these two infective developmental forms of *T. cruzi*, with the ability to induce phagocytosis emerging as a unique property of EAs.

### Phagocytic uptake induced by *T. cruzi* EAs requires intact surface molecules but is not dependent on parasite energy

**Fig. 4:**
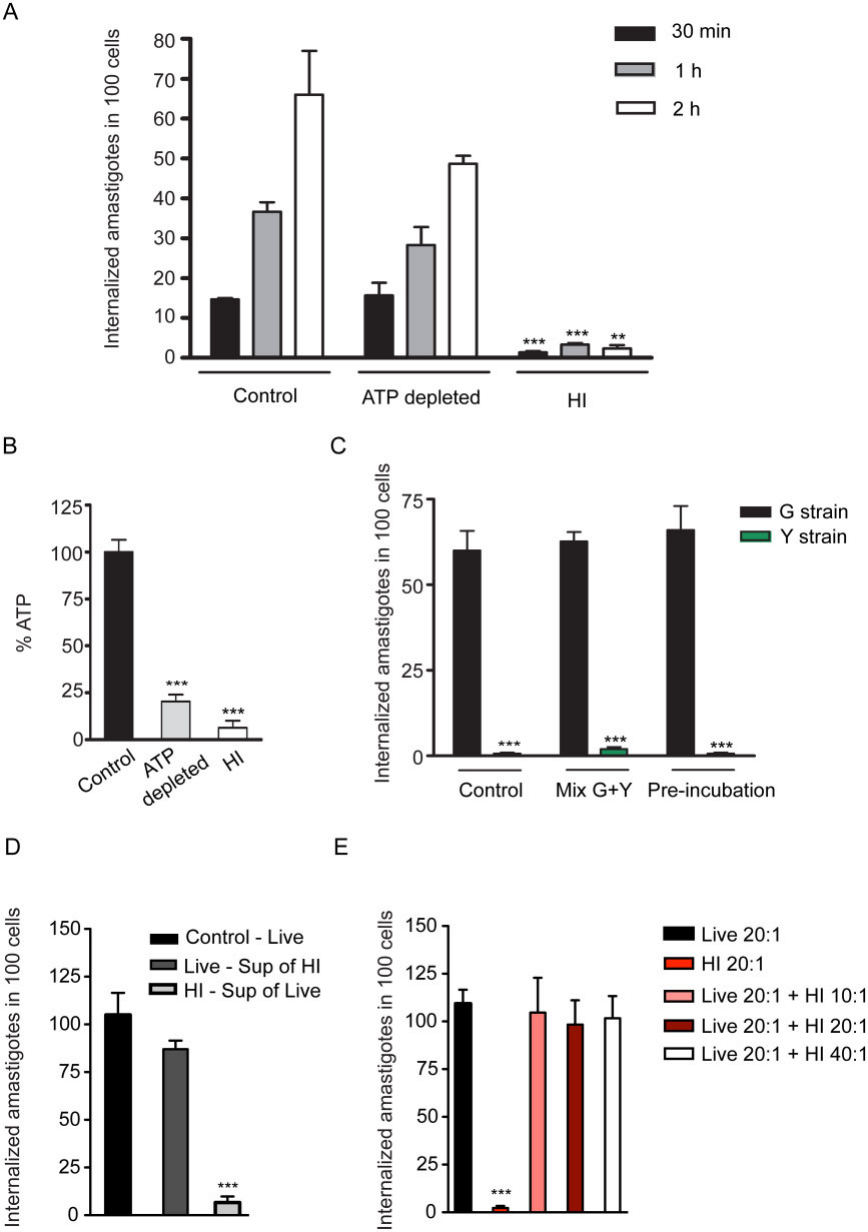
Induced phagocytosis is a property unique to *T. cruzi* G strain EAs, is not dependent on parasite energy or secreted molecules, but requires parasite heat-sensitive surface molecules. A. Invasion kinetics of control, ATP depleted (100 mM sodium azide + 100 mM 2-deoxyglucose) or heat-inactivated parasites (30 min at 56°C). The data correspond to mean of triplicates ± SD. ***P < 0.0001, Student's *t* test; **P = 0.0022, Student's *t* test comparing control and heat-inactivated parasite internalization at the indicated time points. B. ATP levels measured in EAs used in (A). The data correspond to mean of triplicates ± SD. ***P < 0.0002, Student's *t* test comparing control to ATP depleted and to heat-inactivated parasites. C. Invasion of G strain (black bars) and Y strain (green bars) EAs. Y strain EAs were tagged with GFP to allow distinction between the strains. In the control condition, each strain was pre-incubated 2 h in DMEM before exposure to HeLa cells. In Mix G + Y condition, G and Y strain EAs were mixed and pre-incubated for 2 h before simultaneously exposed to HeLa cells. Their invasion efficiency was quantified and plotted separately. In the pre-incubation condition, G strain EAs were exposed to HeLa cells in the presence of conditioned medium from Y strain EAs (2 h pre-incubation), while Y strain EAs were exposed to HeLa cells in the presence of conditioned medium from G strain EAs. Parasites were exposed to cells for 2 h. The data correspond to mean of triplicates ± SD. ***P < 0.0004, Student's *t* test comparing control G strain EA internalization and Y strain internalization. D. Live or heat-inactivated parasites were incubated for 2 h, their supernatants (conditioned medium) were collected and swapped between the samples during exposure to host cells. Black bar: EAs not pre-incubated; dark grey bar: EAs taken up in the presence of heat-inactivated parasites' supernatant; light grey, heat-inactivated EAs taken up in the presence of live parasites' supernatant. The data correspond to mean of triplicates ± SD. ***P < 0.0001, Student's *t* test. E. Live and heat-inactivated parasites were mixed together and exposed to HeLa cells for 2 h at the indicated MOI (pink, maroon and white bars). As control, live (black bar) and heat-inactivated (red bar) parasites were separately exposed to HeLa cells. The data correspond to mean of triplicates ± SD. ***P < 0.0001, Student's *t* test.

To determine if EAs ability to trigger phagocytosis was energy dependent, we performed ATP depletion in the parasites with azide and 2-deoxyglucose (Schenkman *et al*., 1991) and performed invasion assays in HeLa cells, comparing EA internalization efficiency of control and heat-inactivated parasites (Fig. 4A). The parasite ATP levels were also determined, confirming that the treatment resulted in a strong depletion of more than 75% compared with controls (Fig. 4B). The results of infection assays indicated that ATP-depleted EAs were still capable of efficiently triggering phagocytosis in HeLa cells, while the uptake of heat-inactivated parasites performed in parallel was significantly inhibited (Fig. 4A).

It was previously shown that EAs from different *T. cruzi* strains vary in their capacity to enter mammalian cells (Fernandes and Mortara, 2004; Fernandes *et al*., 2007a). In order to determine if secreted molecules from the G strain were involved, we exposed HeLa cells to a mixed population of GFP-tagged Y strain (type II) and untagged G strain (type I) EAs and quantified the number of internalized parasites in HeLa cells under different conditions (Fig. 4C). In control experiments, parasites were pre-incubated for 2 h in DMEM and then exposed to HeLa cells in the presence of their respective supernatant. As expected, Y strain EAs were internalized much less efficiently than G strain EAs (Fig. 4C). In another set of assays, (Mix G + Y), G and Y strain EAs were mixed at a 1:1 ratio and pre-incubated for 2 h before exposure to HeLa cells. Their respective invasion capacity (distinguished by GFP expression – Y strain) was quantified and plotted separately. Interestingly, the presence of the more infective G strain EAs did not influence the level of phagocytosis of the less infective Y strain parasites, indicating that under these conditions, secreted molecules do not play a role in the potent induction of phagocytosis by these parasites (Fig. 4C). In the pre-incubation experimental condition, G strain EAs were exposed to HeLa cells in the presence of conditioned medium prepared with Y strain EAs (2 h pre-incubation), while Y-GFP strain EAs were exposed to HeLa cells in the presence of conditioned medium prepared from G strain EAs (Fig. 4C). In all conditions, the parasites were incubated with host cells for 2 h. In order to determine if molecules with potential inhibitory effects could play a role in EA entry we incubated parasites with HeLa cells in the presence of heat-inactivated parasite supernatant, (Fig. 4D) or in the presence of increasing concentration of heat-inactivated parasites (Fig. 4E). The results demonstrate that supernatant of either live or dead parasites does not affect EA invasion (Fig. 4D), and also that the presence of heat-inactivated parasites during live parasite invasion has no inhibitory effect on the internalization of EA (Fig. 4E). Collectively, the results of these assays clearly indicate that the marked ability of G strain *T. cruzi* EAs to induce phagocytosis requires recognition by host cells of heat-labile molecules associated with the surface of live parasites.

Importantly, unlike what has been demonstrated for trypomastigotes (Schenkman *et al*., 1991; Martins *et al*., 2011) phagocytosis of *T. cruzi* EAs is not dependent on the parasite's ATP stores, but appears to be solely driven by the host cell phagocytic machinery after attachment and stimulation by the parasites.

### EAs engage a molecular machinery similar to the one required by professional phagocytes for the phagocytosis of large particles

Since EA uptake requires host cell actin polymerization and is dependent on surface properties but not on parasite secreted molecules, we hypothesized that EAs might engage a phagocytic machinery that is similar to that utilized by professional phagocytes during large particle phagocytosis. When macrophages take up large particles such as zymosan, the calcium sensor synaptotagmin VII (Syt VII) mediates the delivery of supplemental lysosomal membrane to the nascent phagosome, facilitating particle uptake (Czibener *et al*., 2006). Interestingly, the lysosomal membrane proteins CD63 and Lamp1 are also sequentially recruited to the nascent phagosome (Czibener *et al*., 2006; Flannery *et al*., 2010). The tetraspanin CD63 acts as a trafficking chaperone for the delivery of Syt VII from the Golgi apparatus to lysosomes, explaining why CD63 and Syt VII are delivered simultaneously to phagosomes, preceding the late endosomal/lysosomal resident glycoprotein protein Lamp1 (Flannery *et al*., 2010).

**Fig. 5:**
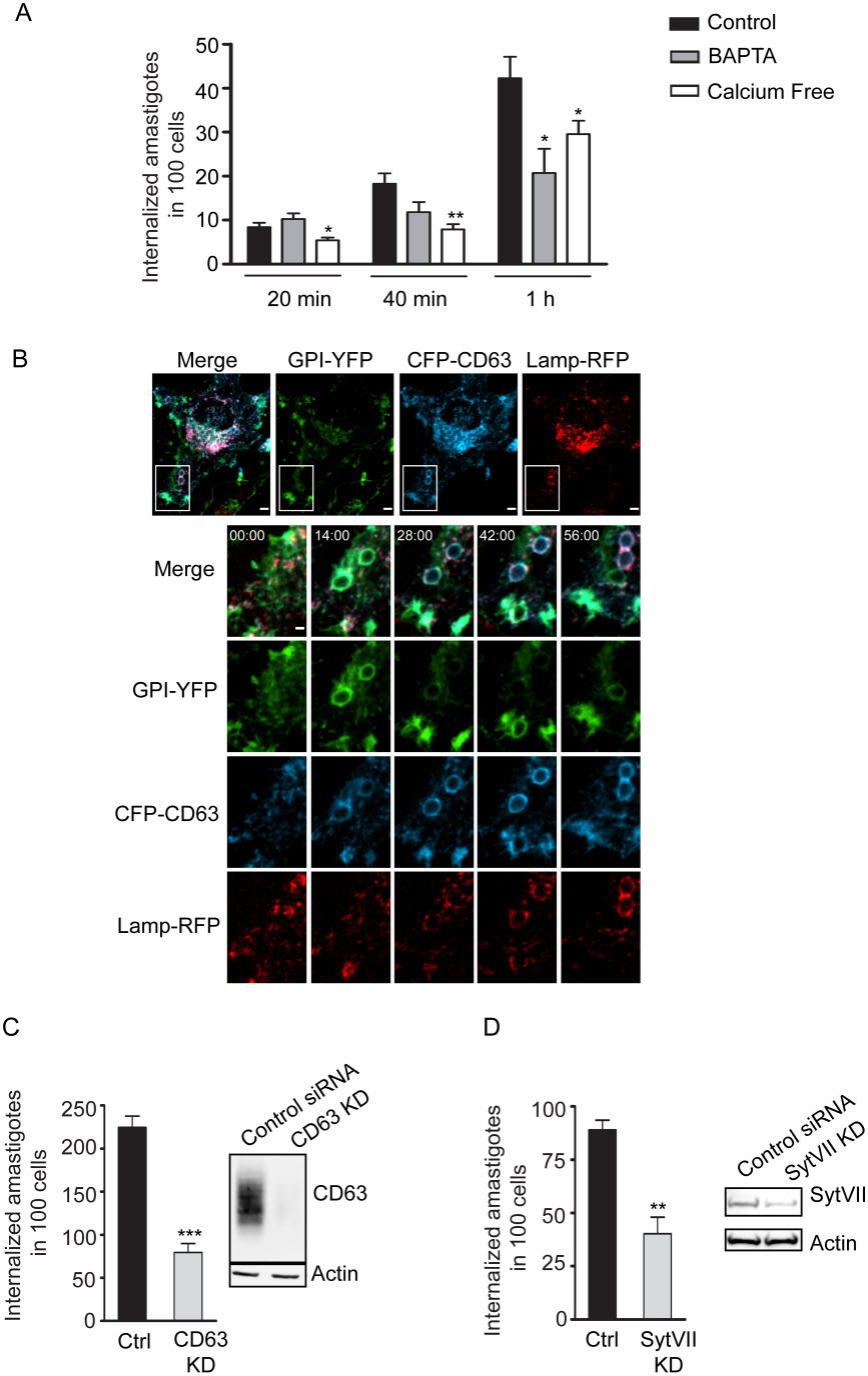
Phagocytosis induced by *T. cruzi* G strain EAs in HeLa cells requires the host lysosomal molecules CD63 and Syt VII, which are recruited to phagosomes before Lamp1. A. Invasion assay with calcium depleting agents: calcium-free DMEM + 5 mM EGTA or 100 μM BAPTA-AM treatment 30 min prior to infection. Uptake assays were performed in calcium-free medium in all conditions. The data correspond to mean of triplicates ± SD. *P < 0.05, Student's *t* test; **P = 0.0087, Student's *t* test comparing control and BPTA treatment or control and calcium-free medium. B. HeLa cells were transduced with GPI-YFP, CFP-CD63 and Lamp-RFP, and infected with G strain EAs (MOI 50:1). Images were acquired by time-lapse spinning-disk confocal microscopy for 56 min at one frame per 2 min. Panels show merged images, GPI-YFP (green), CFP-CD63 (blue), Lamp1-RFP (red) from Video S1 – 56 min. Bars, 5 μm. Lower panels: magnification of the inset highlighted by the white box at 0, 14, 28, 42 and 56 min time points. Bar, 2 μm. C. Internalization of EAs in HeLa cells treated with control or Syt VII siRNA. The data correspond to mean of triplicates ± SD. **P = 0.0028, Student's *t* test comparing control or Syt VII siRNA treatment. D. Internalization of EAs in HeLa cells treated with control or CD63 siRNA. The data correspond to mean of triplicates ± SD. ***P = 0.0005, Student's *t* test comparing control or CD63 siRNA treatment. Invasion period: 2 h for (C) and (D).

To test if calcium-dependent processes played a role in EA invasion, we conducted invasion assays in culture medium with or without calcium. EA uptake by HeLa cells in calcium-free media was significantly reduced compared with medium containing calcium (Fig. 5A). Because internal calcium stores have been shown to play a role in large particle phagocytosis (Czibener *et al*., 2006), and ligation of classic phagocytic receptors results in transient increases in intracellular calcium concentrations (Young *et al*., 1984), we also quantified the entry to EAs in HeLa cells pre-treated with the cell permeant calcium chelator BAPTA. The cells pre-treated with BAPTA showed a significant reduction in the number of internalized parasites (Fig. 5A). These results agree with the hypothesis that *T. cruzi* EAs are internalized through a calcium and lysosome-dependent pathway similar to that engaged by professional phagocytes for efficient phagocytosis of large particles (Braun *et al*., 2004; Czibener *et al*., 2006).

We reasoned that if the same machinery involved in large particle phagocytosis uptake in macrophages was being engaged, then its components should also be recruited to the nascent phagosome during EA entry. To test this, HeLa cells were simultaneously transduced with adenovirus encoding the plasma membrane marker GPI-YFP and the lysosomal markers CFP-CD63 and Lamp1-RFP (Fig. 5B, Video S1). Live time-lapse confocal microscopy showed an initial enrichment of GPI-YFP around EAs moving into the cells, followed by the sequential recruitment of CFP-CD63 and Lamp1-RFP. The parasites were completely surrounded by the plasma membrane marker GPI-YFP on average between 6 and 8 min after contact with the cell. CD63 recruitment to the phagosome could already be seen after 4 min of contact with the host cell, with a complete outline of the parasites coinciding with the time of complete engulfment. Lamp1 and CD63 positive vesicles were visible being recruited to the parasite containing vacuole around 12 min after internalization (Fig. 5B, Video S1). Although slightly slower than large particle phagocytosis by macrophages (Czibener *et al*., 2006; Flannery *et al*., 2010), our data strongly suggested that the overall process of *T. cruzi* EA uptake by HeLa cells was very similar to large particle phagocytosis by macrophages.

Large particle phagocytosis is greatly reduced in Syt VII −/− macrophages, suggesting that the calcium sensor Syt VII plays a role in recruiting lysosomal membrane to the phagosome (Czibener *et al*., 2006). To test if Syt VII was also engaged by the non-professional phagocyte machinery during EA engulfment, siRNA-mediated silencing of either Syt VII or CD63 was performed in HeLa cells. The siRNA-treated cells displayed a marked reduction in the number of internalized EAs compared with control cells (Fig. 5C and D), indicating that lysosomal recruitment to the phagosome is also likely to be involved in enhancing EA uptake. Together, these data support our hypothesis that *T. cruzi* EAs mobilize machinery similar to that used by professional phagocytes.

### The dynamics of lipid metabolism in phagosomes containing EAs in HeLa cells resembles the pattern seen in macrophages

Phagocytosis can be triggered by several unique phagocyte receptors, ultimately resulting in the engulfment of the particle or pathogen. The initiation of phagocytosis involves lipid modification, secondary messenger generation and redistribution of key proteins (Yeung and Grinstein, 2007). Extensive work on FcR-mediated phagocytosis revealed that phosphoinositide lipids play a key role in regulating early events in phagosome formation. Phosphoinositides contain an inositol ring that can be phosphorylated at position 3, 4 or 5, generating up to six unique molecules. At the start of phagocytosis, [PI(4,5)P_2_] undergoes a transient accumulation at the base of the forming phagosome and influences actin remodelling (Botelho *et al*., 2000; Takenawa and Itoh, 2001). Shortly after cup formation, PI(4,5)P_2_ hydrolysis occurs generating diacylglycerol and inositol-1,4,5-triphosphate, which is a potent signal for calcium store mobilization (Yeung and Grinstein, 2007). PI(4,5)P_2_ can also be phosphorylated by phosphoinositide 3-kinase to produce [PI(3,4,5)P_3_]. Hydrolysis or phosphorylation lead to PI(4,5)P_2_ removal from the nascent cup, a process that shown to be required for successful completion of phagocytosis (Yeung and Grinstein, 2007). On the other hand, the transient biogenesis of PI(3,4,5)P_3_ is essential for extension and fusion of the nascent cup around the ingested particle, and is inducible within 2–3 min of phagosomal sealing in FcR-mediated phagocytosis (Marshall *et al*., 2001). This marked but short-lived burst of PI(3,4,5)P_3_ recruits protein kinases, adaptor proteins and guanine nucleotide exchange factors to activate small GTPases, which are important for phagosome development (Marshall *et al*., 2001; Botelho and Grinstein, 2011). Finally, phosphatidylinositol-3-phosphate [PI(3)P] is found on early endosomes and newly formed phagosomes at about 1 min after phagosome sealing, leading to phagosome maturation (Fratti *et al*., 2001; Vieira *et al*., 2001).

**Fig. 6:**
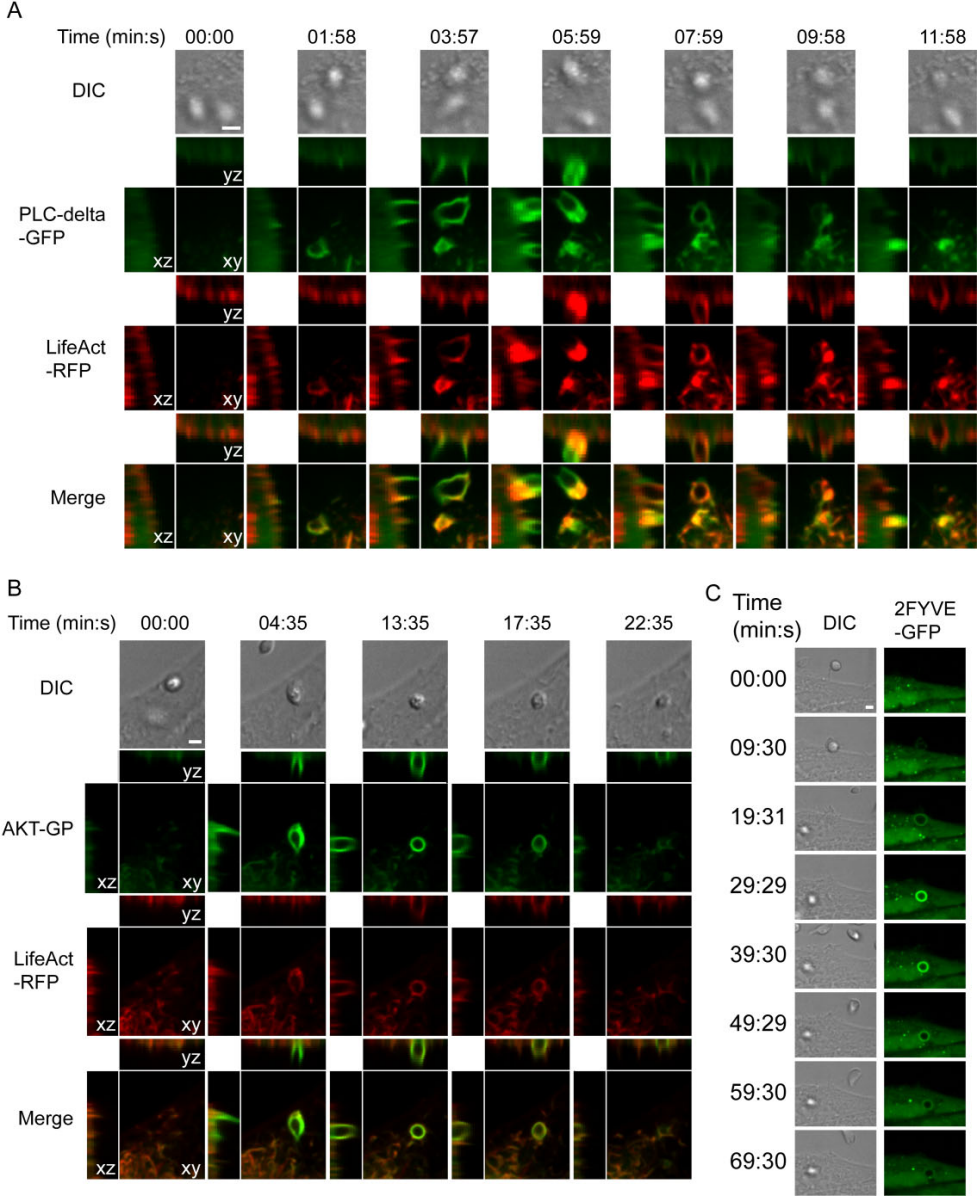
The phosphoinositide markers PH-PLC∂, PH-AKT and FYVE domain are sequentially recruited to nascent phagosomes containing *T. cruzi* G strain EAs, with a slower kinetics than what was previously shown for zymosan particles in macrophages. HeLa cells were transfected with plasmids encoding PH-PLC∂-GFP, PH-AKT-GFP or 2-FYVE-GFP together with LifeAct-RFP, and infected with *T. cruzi* G strain EAs (MOI 50:1). A. Images were acquired by time-lapse spinning-disk confocal microscopy for 16 min at one frame per 30 s. B. Images were acquired by time-lapse spinning-disk confocal microscopy for 41 min at one frame per 30 s. C. Images were acquired by time-lapse spinning-disk confocal microscopy for 90 min at one frame per 30 s. A–C. Panels show differential interference contrast (DIC), PI-binding-domain-GFP (green), LifeAct-RFP (red) and merged fluorescence images. For the fluorescence images, the left panels represent the X–Z plane, the top panel represents the Y–Z plane, and the middle panel represents the X–Y plane centred at the amastigote. Time is displayed in minutes : seconds. Bars, 1 μm.

To test whether EA-containing phagosomes sequentially acquired and lost phosphoinositide phagocytosis reporter molecules during internalization, we followed phosphoinositide metabolism during parasite invasion with GFP-labelled probes and live cell spinning disk confocal microscopy. The first lipid to be enriched at the nascent phagosome was PI(4,5)P_2_, which can be monitored with the PH domain from phospholipase C ∂ (PH-PLC∂) (Botelho *et al*., 2000). HeLa cells were transfected with plasmids encoding PH-PLC∂-GFP and LifeAct-RFP (a 17-amino-acid peptide that labels F-actin) and exposed to G strain EAs. After contact of the parasite with the cell, phagocytosis was initiated as seen by PH-PLC∂-GFP and actin recruitment (Fig. 6A, Video S2). This indicated that PI(4,5)P_2_ was enriched at the site of phagocytosis and the marker, PH-PLC∂-GFP, was retained at the base of the nascent phagosome for approximately 7–8 min and then lost as the parasite was internalized. To follow the next stage of phosphoinositide production, HeLa cells were transfected with constructs encoding the PH domain of AKT fused with GFP, which binds to PI(3,4,5)P_3_ and LifeAct-RFP. As expected, PI(3,4,5)P_3_ monitored by PH-AKT-GFP began to be recruited approximately 4 min after the beginning of phagocytosis and persisted on the nascent phagosome for 20–30 min (Fig. 6B, Video S3). This was an interesting observation, since it contrasts with the fast PI(3,4,5)P_3_ disappearance that was reported to occur as early as 1 min after phagosome sealing in macrophages (Vieira *et al*., 2001), coinciding with loss of the YFP-GPI signal resulting from quenching in an acidic environment (Fig. 4, Video S1). Following phagosome sealing in macrophages, PI(3)P becomes apparent on sealed phagosomes (Vieira *et al*., 2001). To monitor PI(3)P levels in HeLa cells, a plasmid encoding two tandem FYVE domains fused to GFP (2FYVE-GFP) were transfected into HeLa cells and exposed to G-strain EAs. Approximately 20–30 min after initiation of phagocytosis, the nascent phagosome recruited 2FYVE-GFP indicating the formation of PI(3)P (Fig. 6C, Video S4). The signal persisted for approximately 50–70 min until it dissipated. This timing was significantly longer than what is observed in classic phagocytosis by macrophages, with PI(3)P persisting for approximately 10 min after phagosome sealing (Yeung and Grinstein, 2007). Thus, while the kinetics of the PI metabolism appears to be delayed when compared with professional phagocytes, the same sequential events are in place.

**Fig. 7:**
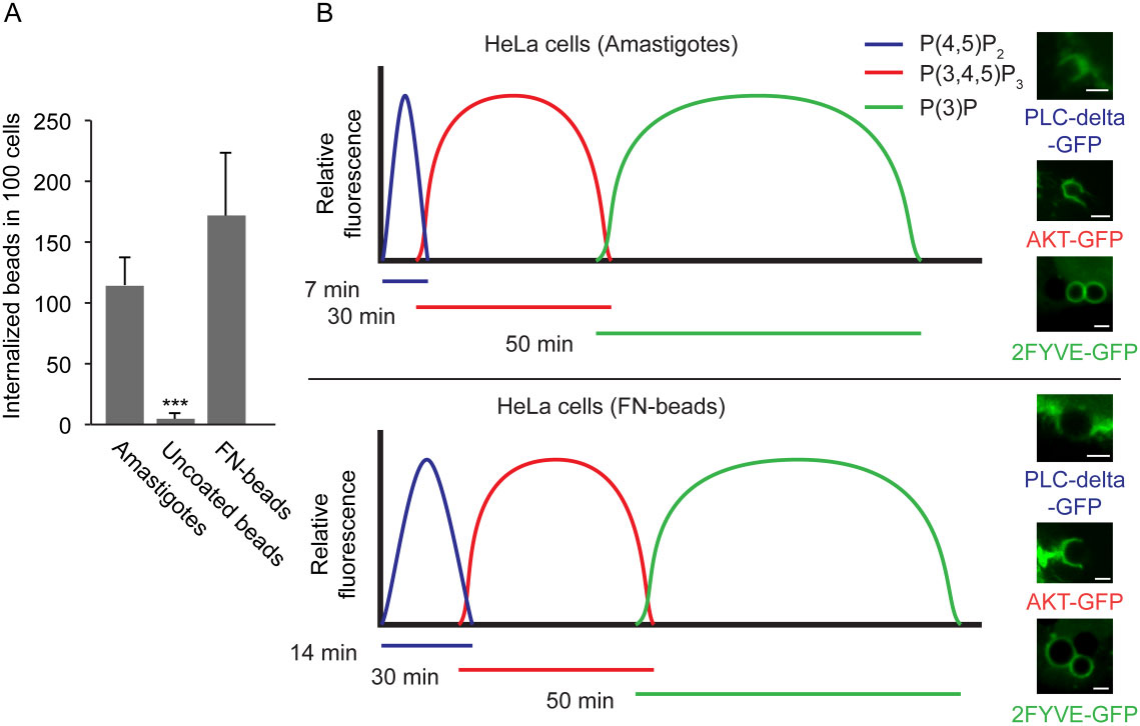
EAs display similar uptake kinetics as fibronectin-coated latex beads. A. HeLa cells were incubated with EAs (Amastigotes), fibronectin-coated beads (FN-beads) or uncoated beads (ratio 20:1) and exposed to HeLa cells for 2 h. The data correspond to mean of triplicates ± SD. ***P = 0.0005, Student's *t* test. B. HeLa cells were transfected with plasmids encoding PH-PLC∂-GFP, PH-AKT-GFP or 2-FYVE-GFP together with LifeAct-RFP, and infected with *T. cruzi* G strain EAs (MOI 50:1) or fibronectin-coated beads (ratio 3:1). The timing of lipid metabolism was recorded as the resident time of lipid markers on the phagosomal membrane, based on the relative abundance of PI(4,5)P_2_ (blue), PI(3,4,5)P_3_ (red) and PI(3)P (green). The curves for the *T. cruzi* G strain and fibronectin-coated beads represent idealized profiles derived from experimental data with heights reflecting relative changes and not absolute concentrations. Representative images are shown to the right of the graphs for *T. cruzi* G strain and fibronectin-coated beads.

In order to determine if the delayed kinetics in the recruitment of 2FYVE-GFP in HeLa cells reflects an intrinsic characteristic of non-professional phagocytes, we compared the uptake of EAs to the internalization of inert particles. As expected, coating latex beads with fibronectin (FN-beads) (Ozeri *et al*., 1998) promoted uptake at levels comparable to EAs, while uncoated beads showed insignificant uptake (Fig. 7A). Comparison of uptake kinetics of FN-beads and EA revealed that internalization process in HeLa cells, in both cases, presents a delay in marker recruitment (Fig. 7B) when compared with macrophages (Yeung and Grinstein, 2007). This result strongly suggests that while epithelial cells contain the machinery for particle uptake by phagocytosis, the duration of the process is significantly extended. Interestingly, our results also revealed that during EA-induced phagocytic cup formation, the PI(4,5)P_2_ recruitment period is longer, when compared with inert particles (Fig. 7B). However, it has been demonstrated that the geometry of the phagocytosed particle greatly influences phagocytic responses (Doshi and Mitragotri, 2010), and differences in shape between round latex beads and the oval EAs may account for this variation. Altogether our results reveal that although the kinetics seems to be delayed, non-professional phagocytes are capable of classical phagocytosis and that *T. cruzi* EAs are not responsible for delaying the process.

Although the precise role of extracellularly generated amastigotes in the pathogenesis of Chagas' disease has only been hypothesized (Scharfstein and Morrot, 1999) it is conceivable that the potent phagocytic properties of *T. cruzi* amastigotes play a role in parasite persistence within the mammalian host. Immune lysis of infected cells is predicted to release viable amastigotes into the extracellular medium, and a potent induction of re-uptake by nearby cells can promote parasite persistence in tissues, a hallmark of the chronic infection that is characteristic of Chagas' disease.

## Experimental procedures

### Host cells and parasites

HeLa cells CCL-2.1 (ATCC), NRKs, C2C12 and LLC-MK_2_ were grown in Dulbecco's modified Eagle's medium (DMEM – Sigma) supplemented with 10% of fetal bovine serum (FBS), at 37°C with 5% CO_2_. Trypomastigotes from *T. cruzi* G strain (Yoshida, 1983) were obtained from the supernatant of infected monolayers of HeLa cells as previously described (Fernandes *et al*., 2007b). Trypomastigotes from the *T. cruzi* Y strain were obtained from the supernatant of infected monolayers of LLC-MK_2_ cells, as previously described (Andrews *et al*., 1987). For EA differentiation, cell derived trypomastigotes (Y or G strain) were isolated from culture supernatants of infected cells by centrifugation at 1200 *g* for 10 min. The pellet was resuspended in LIT medium, pH 5.8 and incubated for 24 h at 37°C, and at least 95% pure EAs were obtained (Ley *et al*., 1988; Mortara, 1991).

Y strain-GFP parasites were obtained after transfection of p-TREX–GFP vector (DaRocha *et al*., 2004) in epimastigotes forms of *T. cruzi*. Briefly, epimastigotes were harvested from cultures grown in liver infusion tryptose (LIT) medium containing 10% FBS at 28°C (Nogueira and Cohn, 1976), washed once with PBS and resuspended to 2 × 10^8^ parasites ml^−1^ in electroporation buffer (137 mM NaCl, 21 mM HEPES, 5 mM KCl, 5.5 mM Na_2_HPO_4_, 0.77 mM glucose, pH 7.0). Aliquots (0.7 ml) of parasite suspension were mixed with 25 μg of DNA in 0.4 cm cuvettes and electroporated using a Bio-Rad Gene Pulser® set at 0.3 kV and 500 μF with two pulses. The transfected cells were transferred to 5 ml of LIT with 10% FCS and incubated at 28°C for 48 h before adding G418 (500 μg ml^−1^). Plasmid DNA used in electroporation experiments was obtained by alkaline lysis using Qiagen columns (Qiagen, USA).

### Cell invasion assays

Cells (1.8 × 10^5^ per well) were plated on glass coverslips placed in 35 mm wells, 24 h before experiments. EAs were washed twice with phosphate-buffered saline (PBS) and resuspended in 10% FBS–DMEM and incubated for 1 h at 37°C to recover from differentiation medium. Semiconfluent cells were then infected with G strain EAs (MOI 15:1) or Y strain EAs (MOI 50:1) in 2% FBS–DMEM for the indicated period of times.

For calcium chelation, invasion assays were performed in calcium-free DMEM (Invitrogen) in all conditions. HeLa cells were pre-treated for 30 min prior invasion with either DMEM containing 5 mM EGTA, or calcium-free DMEM containing 100 μM BAPTA-AM (Invitrogen). Samples were fixed for 5 min at room temperature with Bouin solution (71.4% saturated picric acid, 23.8% formaldehyde and 4.8% acetic acid), stained with Giemsa and sequentially dehydrated in acetone followed by a graded series of acetone : xylol (9:1, 7:3, 3:7) and finally xylol. This technique allows microscopic distinction between intracellular parasites, which are seen surrounded by a halo, from attached parasites. The number of intracellular parasites was determined by counting at least 300 cells per coverslip, in triplicate in a Nikon E200 microscope with a 100× N.A. 1.3 oil immersion objective. For immunofluorescence, coverslips were fixed in 4% paraformaldehyde (PFA) diluted in PBS for 15 min and processed as described below. For inert particle internalization assays, polystyrene beads (Spherotech, 5 μm) were washed three times with cold PBS without calcium or magnesium (PBS −/−). After the final wash, the beads were resuspended in 1 ml of PBS −/− at 5 × 10^7^ beads ml^−1^ with 50 μg ml^−1^ fibronectin (BD Biosciences) and incubated overnight at 4°C. The beads were then washed three times with PBS (−/−) and incubated in 1 ml of BSA (10 mg ml^−1^) for 3 h at 4°C. The beads were washed three times with cold PBS −/− and suspended in DMEM culture media for use in uptake assays and live cell microscopy. Zymosan was purchased from Sigma.

### Immunofluorescence

PFA fixed cells were washed with PBS, quenched with 15 mM NH_4_Cl for 15 min, and incubated with PBS containing 2% bovine serum albumin (BSA) for 1 h. When processed for an inside/outside immunofluorescence assay (Tardieux *et al*., 1992) samples were incubated with rabbit anti-*T. cruzi* polyclonal antibodies for 1 h followed by 1 h anti-rabbit IgG conjugated to AlexaFluor™ 647 (Invitrogen, for confocal images) or AlexaFluor™ 594 (for quantification) secondary antibodies, to stain extracellular parasites. Cells were then permeabilized with 0.1% saponin for 30 min and incubated with H4A3 anti-human Lamp1 mouse monoclonal antibodies. (Developmental Studies Hybridoma Bank) for 1 h followed by 1 h incubation with anti-mouse AlexaFluor™ 488 secondary antibodies and Phalloidin-594 (Invitrogen) to stain actin filaments. The images shown in Fig. 1 (all panels) maximum projections images of optical sections (0.13 μm Z step) acquired on a Leica SPX5 confocal system with a 63× N.A. 1.4 oil objective.

In order to quantify internalized G strain or Y strain-GFP EAs or inert particles (Fig. 3C), inside/outside fluorescence assay was performed as described above and slides were analysed using Nikon E200 epifluorescence microscope with a 100× N.A. 1.3 oil immersion objective. The number of internalized parasites (negative for anti-*T. cruzi* antibody staining) was determined in at least 300 cells. All samples were incubated with 10 μM DAPI (Sigma) for nuclei and kinetoplasts staining.

### ATP depletion, ATP assays and heat inactivation

For ATP depletion, parasites were treated with 100 mM sodium azide and 100 mM 2-deoxyglucose in glucose free DMEM (Gibco) for 1 h (Schenkman *et al*., 1991). Invasion assays were performed subsequently for the indicated time period.

Parasites were washed twice with PBS and resuspended at 1e10^5^ parasites per 100 μl. Amount of ATP was determined with the ATPlite assay kit (PerkinElmer) as outlined by manufacturer's protocol. The data were acquired on SpectraMax m5e plate reader (Molecular Devices) and analysed with the Softmax Pro software package (Molecular Devices). Parasites were heat-killed at 56°C in a water bath for 20 min.

### Electron microscopy

HeLa cells were grown in glass coverslips and allowed to interact with G strain EAs at a 10–20:1 parasites per cell ratio. After 30 min when parasites are mostly initiating contact, cells were washed with 0.1 M phosphate-buffered saline (37°C) five times to remove unattached parasites and fixed with 2% glutaraldehyde, 4% paraformaldehyde (both from Electron Microscopy Sciences – EMS, Hatfield, PA, USA), 140 mM NaCl, 2 mM CaCl_2_, 1 mM MgCl_2_ in 0.1 M HEPES (pH 7.2) for 30 min. To reveal cytoskeletal elements, after 30 min of interaction, samples were treated with a membrane extraction solution containing 1% Triton X-100, 100 mM PIPES (pH 7.2), 4% sucrose, 1 mM MgCl_2_, 10 μM taxol (Invitrogen) and 10 μM phalloidin (Sigma) – to stabilize microtubules and microfilaments respectively (Sant'Anna *et al*., 2005) – for 10 min under gentle rocking at room temperature then washed twice for 10 min in the same solution without the detergent, then fixed as described above. Samples were post-fixed with 1% OsO_4_ (EMS) and 1% tannic acid (EMS), both for 1 h, dehydrated with ethanol and critical point-dried from CO_2_ (CPD 020 Balzers-Tec). The slides were coated with a thin carbon layer (8 nm) (BAF 300, Balzers) and the glass coverslips detached from the cell layer by immersion in 20% hydrofluoric acid (Sigma). Small pieces of the cell layer were transferred for distilled water for 10 min and finally collected onto copper grids (300 mesh). Images were acquired on a Hitachi S-4800 field emission scanning electron microscope operated at 5 kV.

### Plasmids and adenoviral constructs

Constructs encoding the PH domain of PLC-δ fused to GFP (PLC-δ-GFP), the PH domain of Akt fused to GFP (AKT-GFP) and the tandem FYVE domains fused to GFP (2FYVE-GFP) were described previously (Stauffer *et al*., 1998; Haugh *et al*., 2000; Vieira *et al*., 2001). These plasmids were generous gifts from Sergio Grinstein (Program in Cell Biology, The Hospital for Sick Children, 555 University Avenue, Toronto, Ontario M5G 1X8, Canada). The mammalian expression vector encoding a 17-amino-acid peptide fused to TagRFP, pCMVLifeAct-TagRFP (LifeAct-RFP) for labelling actin filaments was purchased from ibidi. The adenoviral production and purification of virons encoding CFP-CD63, Lamp-RFP and GPI-YFP were described previously (Keller *et al*., 2001; Flannery *et al*., 2010).

### Transfection, adenoviral transduction, live cell imaging and image analysis

Before live cell imaging experiments, HeLa cells were seeded at 1.8 × 10^5^ cells per dish in DMEM media at 37°C and 5% CO_2_ in a 35 mm glass-bottom dish (MatTek Corporation). For adenoviral transduction, cells were transduced for 18 h by replacing the culture media with HeLa imaging medium (DMEM without phenol red, 10% FBS, 1% penicillin/streptomycin) and containing purified adenovirus encoding CFP-CD63, Lamp-RFP and GPI-YFP at a 10:1 MOI. For plasmid transfection, cells were transfected with plasmids encoding either PLC-δ-GFP, AKT-GFP or 2FYVE-GFP and LifeACT-RFP with Fugene HD (Roche) according to the manufacturer's protocol. After 18 h, the cells were washed, medium containing G strain EAs (50:1 MOI) was added, and the dishes were imaged.

For imaging, dishes were placed in a LiveCell System chamber (Pathology Devices) at 37°C with 5% CO_2_ attached to an Eclipse Ti inverted microscope with a 60× NA 1.4 objective (Nikon). Spinning disk confocal images were acquired using the UltraVIEW VoX system (PerkinElmer) equipped with a Hamamatsu C9100-50 camera, analysed and edited using the Volocity Software Suite (PerkinElmer). Final image gamma settings were changed to 1.4.

### CD63 and Syt VII transcriptional silencing

HeLa cells were transfected with Lipofectamine RNAiMAX and 160 pmol of medium GC content control (12935300), CD63 (HSS101615) or Syt VII (HSS113401) Stealth siRNA duplexes, according to the manufacturer's instructions (Invitrogen). At 24 h after transfection, the cells were submitted to invasion assay.

### Western blot analysis

To generate whole-cell lysates, HeLa cells treated with siRNA as outlined above, were lysed in WCL buffer (25 mM HEPES pH 7.4, 150 mM NaCl, 1% Triton X-100 and complete protease inhibitor cocktail from Roche) at 4°C for 1 h. Lysates were clarified by centrifugation at 10 000 *g* for 10 min, and the total amount of protein was determined by BCA (Thermo Scientific). A volume of clarified lysate containing 20 μg of protein was added to 6× SDS sample buffer, resolved on 10% SDS-PAGE, transferred to nitrocellulose, and probed with either rabbit anti-Syt VII (Arantes and Andrews, 2006), mouse anti-CD63 antibodies (H5C6, 1:200) or mouse anti-β actin (Sigma, 1:1000). Blots were then incubated with either peroxidase-conjugated donkey anti-rabbit or donkey anti-mouse antibodies (Jackson ImmunoResearch, 1:10000), washed, incubated for 5 min with Immun-Star HRP™ solution (Bio-Rad), and visualized on a LAS-3000 imaging system (Fujifilm). Relative protein levels were determined using the Image-J software (NIH).

### Statistics

All experiments were performed in triplicate coverslips and repeated at least three times, and 30 cells per coverslip were counted. Statistical analysis was performed with GraphPad Prism®, employing Student's *t* test. Data are presented as mean ± standard deviation (SD).

## Supporting information

**Video S1.** Sequential recruitment of plasma membrane GPI-YFP and lysosomal CD63 and Lamp1 to nascent phagosomes containing *T. cruzi* G strain EAs. HeLa cells were transduced with GPI-YFP, CFP-CD63 and Lamp-RFP and infected with EAs (MOI 50:1). Images were acquired by time-lapse spinning-disk confocal microscopy for 56 min at one frame per 2 min. Individual panels show merged images, GPI-YFP (green), CFP-CD63 (blue), Lamp1-RFP (red). The video is displayed at five frames per second. Bar, 5 μm.

**Video S2.** Recruitment of PH-PLC∂ to nascent phagosomes containing *T. cruzi* G strain EAs. HeLa cells were transfected with a plasmid encoding PH-PLC∂-GFP and LifeAct-RFP, and infected with EAs (MOI 50:1). Images were acquired by time-lapse spinning-disk confocal microscopy for 16 min at two frames per min. Panels show differential interference contrast (DIC), PH-PLC∂-GFP (green), LifeAct-RFP (red) and merged images. For panels displaying fluorescence, the left panels represent the X–Z plane, the top panel represents the Y–Z plane, and the middle panel represents the X–Y plane centred at the amastigote. The video is displayed at five frames per second. Bar, 2 μm.

**Video S3.** Recruitment of PH-AKT to nascent phagosomes containing *T. cruzi* G strain EAs. HeLa cells were transfected with plasmid encoding PH-AKT-GFP and LifeAct-RFP, and infected with EAs (MOI 50:1). Images were acquired by time-lapse spinning-disk confocal microscopy for 41 min at one frame per 30 s. Panels show differential interference contrast (DIC), PH-AKT-GFP (green), LifeAct-RFP (red) and merged images. For panels displaying fluorescence, the left panels represents the X–Z plane, the top panel represents the Y–Z plane, and the middle panel represents the X–Y plane centred at the amastigote. The video is displayed at five frames per second. Bar, 2 μm.

**Video S4.** Recruitment of FYVE domain to nascent phagosomes containing *T. cruzi* G strain EAs. HeLa cells were transfected with plasmid encoding 2-FYVE-GFP and LifeAct-RFP, and infected with EAs (MOI 50:1). Images were acquired by time-lapse spinning-disk confocal microscopy for 90 min at one frame per 30 s. Panels show differential interference contrast (DIC), 2-FYVE-GFP (green), LifeAct-RFP (red) and merged images. The video is displayed at five frames per second. Bar, 2 μm.
